# Distal-continual colon interposition for esophageal reconstruction after esophagectomy: Two case reports

**DOI:** 10.3389/fsurg.2023.1098583

**Published:** 2023-01-30

**Authors:** Bin Guo, Ming He, Jidong Zhao, Minting Ma, Zhanjie Gao

**Affiliations:** ^1^ Department of Thoracic Surgery, Fourth Hospital of Hebei Medical University, Shijiazhuang, China; ^2^ Department of Medical Oncology, Fourth Hospital of Hebei Medical University, Shijiazhuang, China

**Keywords:** esophagectomy, transverse colon, surgical anastomosis, double primary carcinoma of esophagus and heart, case reports

## Abstract

**Background:**

Colon interposition is a complex and time-consuming procedure requiring at least three or four digestive anastomoses. However, the long-term functional outcomes are promising, with an acceptable operative risk.

**Case presentation:**

Herein, two cases of esophageal carcinoma that received esophagus reconstruction using the distal continual colon interposition technique have been described. The transverse colon was lifted to the thoracic cavity for the end-to-side anastomosis with the esophagus, and a closure device was used to close the colon instead of severing and isolating the distal end. The duration of the operation was 140 and 150 min, respectively. The blood supply of the colon was maintained during the intervention. The tension-free anastomosis was performed without severe complications, and oral food intake was resumed on postoperative day 6. Neither anastomotic stenosis, antiacid or heartburn, dysphagia, or emptying obstacles nor complaints of diarrhea, bloating, or malodor were reported during the follow-up period.

**Conclusions:**

The modified distal-continual colon interposition technique may have the advantages of a short operation time and potential prevention of serious complications caused by the torsion of mesocolon vessels.

## Introduction

Colon interposition refers to the application of the colon as an esophageal graft in esophageal and cardiac cancer surgery (1, 2). Colon transplantation is the preferred method after tubular stomach for double primary carcinoma of esophagus and cardia, double primary carcinoma of esophagus and stomach, esophageal cancer after subtotal gastrectomy, and tubular stomach necrosis after replacement (3–5). Compared to the stomach and jejunum, the colon has regular contraction and can provide sufficient length as a graft (6). Most studies recommend the interposition of the transverse colon and part of descending colon along the peristaltic direction with blood supply from the left colon artery (7, 8). Although it was previously demonstrated that using the colon as a graft can adequately achieve food satisfaction and quality of life (8, 9), the procedure has room for improvement.

In most procedures, the transplants are separated from the output colon at the anal side (2). Complete detachment not only increases the difficulty of the operation, prolonging the operation time but also impairs the neurophysiological function of the colon (1, 5). Moreover, the isolated mesentery is prone to torsion wthe transplants are separated from the output colon at the anal side In the Fourth Medical College of Hebei Medical University, colon interposition has become the second choice after radical resection for esophageal cancer, and some modifications were proposed to shorten the operation time and improve the post-surgery prognosis. Instead of severing and isolating the distal end, the transverse colon was lifted to the thoracic cavity for the end-to-side anastomosis with the esophagus, and a closure device was used distal to the anastomosis between the stomach and transverse colon to close the colon.

Herein, two cases of esophageal carcinoma that underwent esophagus reconstruction using the distal continual colon interposition technique modified by authors have been described.

## Case presentation

### Case 1

A male patient, 58-years-old, was diagnosed with double primary carcinoma of the cardia and middle thoracic esophagus. The patient was hospitalized at the Fourth Medical College of Hebei Medical University for further treatment after two cycles of neoadjuvant chemotherapy for cardiac cancer on May 27, 2018. The chemotherapy regimen included oral administration of oxaliplatin 150 mg combined with teggio. The patient visited our hospital on April 22, 2018. Gastroscopy showed smooth esophageal mucosa, the tooth line about 40 cm away from the incisor, and the cardia with small, curved neoplasia, reaching the gastric body and gastric incisura, where the mucosa was damaged. Gastroscopic pathology revealed poorly differentiated adenocarcinoma. The physical examination noted no other significant pathology. On June 7, 2018, the patient underwent proximal gastrectomy with colon interposition through the median, upper abdominal incision; the operation duration was 255 min.

**Figure 1:**
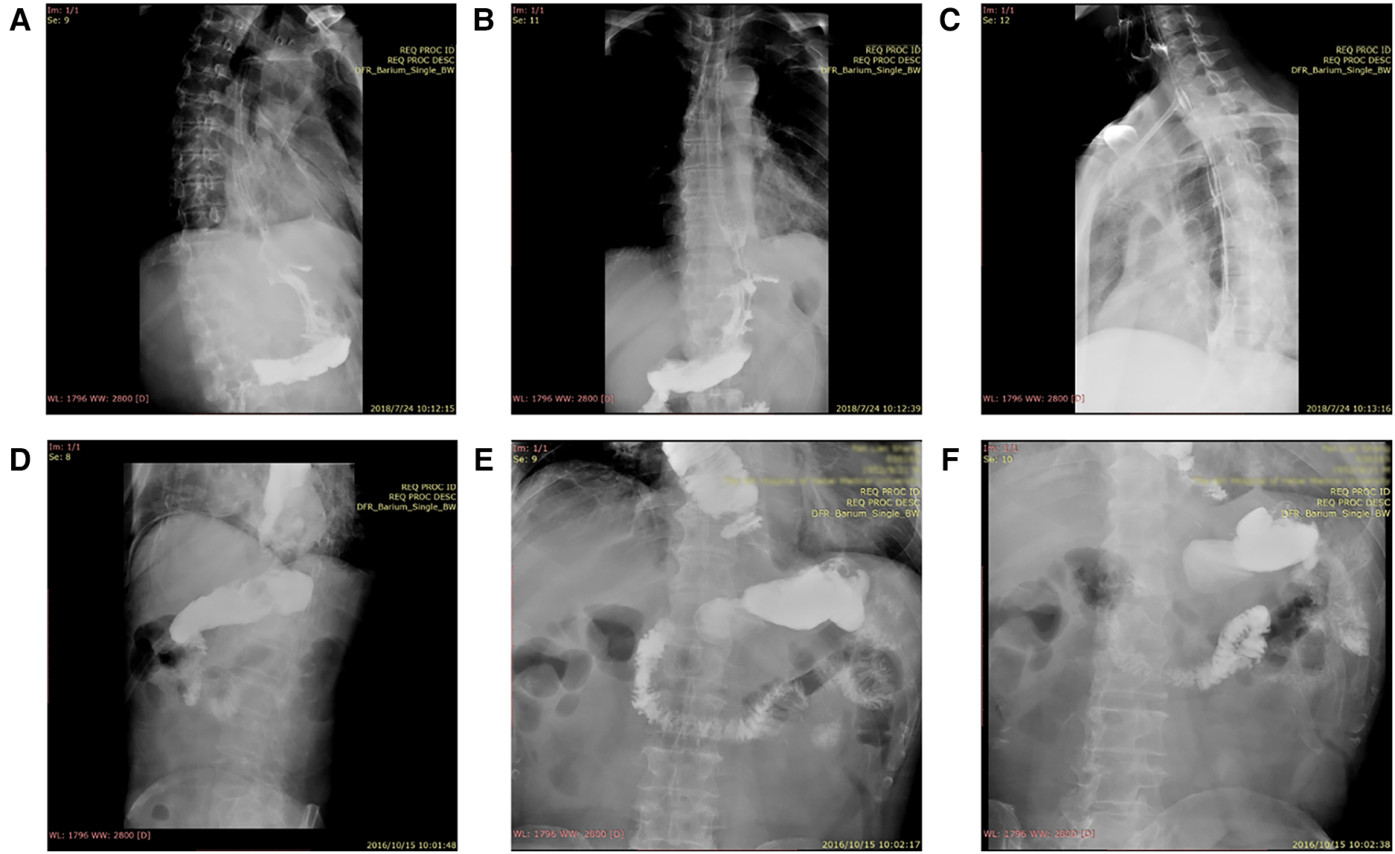
Results of digestive tract angiography postoperative. Patient 1 (**A–C**); Patient 2 (**D–F**).

The specific surgical procedures included tumor resection with the following colon interposition. The operation used two incisions in the right chest and upper abdomen. First, the stomach (preserving the right gastroepiploic artery) was freed through the abdominal cavity, and then the proximal stomach was removed, including the tumor, followed by freeing the transverse colon and the part of the descending colon, selecting the transverse colon as the interposition colon, disconnecting the right end of the transverse colon, ligating the middle colon artery, and preserving the left colon artery to ensure the integrity of the free colon mesentery. Subsequently, the ascending and descending colon, as well as the distal stomach and the output of the transverse colon were anastomosed end-to-side. Both of them used the Circular stapler (CDH25A, ETHINCON, USA). Instead of disconnecting the interposition colon, the transverse colon near the output end of the distal gastric transverse colon anastomosis was ligated with two non-absorbable mousse suture and with staplers (TX60B, ETHICON, USA) at 5-cm distal to the anastomotic opening of the stomach and transverse colon. This closure or ligation was only aimed at the intestinal tube (blocking the passage of the intestinal tube) and did not involve the blood supply of the intestinal tube and the surrounding nerves. Next, the patients were turned over, and the right chest was opened to free the thoracic esophagus. Then, most of the thoracic esophagus, including the tumor, was removed, the transverse colon was lifted to the chest, and end-to-side anastomosis was performed with the esophagus (along peristalsis) using the Circular stapler (CDH23A, ETHINCON, USA). The interposition colon was placed on the posterior mediastinal esophageal bed. Manual suturing was required to suspend the transverse colon seromuscular layer and diaphragmatic Angle for three to four stitches. Systematic lymph node dissection is necessary. The transplanted colon was of appropriate length, rich in blood supply, and underwent anastomosis without tension; the operation duration was 140 min. The patient was transferred to the intensive care unit (ICU) for comprehensive treatment on June 11, 2018. Postoperative intermittent fever did not exceed the maximum temperature of 38.8 °C; a comprehensive treatment was used to prevent infection and stabilize blood pressure. On June 23, 2018, the patient was transferred back to the general ward and given anti-inflammatory fluid infusion and enteral nutrition support. On July 24, 2018, gastrointestinal radiography was performed. The results suggested that the esophagus gastric arch anastomosis was smooth, with 0.8–1.0 cm width, the contrast agent passed smoothly, and no niche and filling defect was found in the residual esophagus (Figures 1A–C). During the follow-up period, the patient had a moderate food intake and weight gain and no acid reflux, nausea, or malodor in the mouth. At the last follow-up on June 1, 2022, no disease progression was observed, and the patient had a satisfactory quality of life.

### Case 2

A 64-years-old male patient was hospitalized in the Fourth Medical College of Hebei Medical University on October 6, 2016, due to eating problems that lasted for 1 month. The patient has a 10-year history of controlled hypertension and a 5-year history of coronary heart disease but no history of drug and food allergies. The results of electronic gastroscopy showed that the esophageal mucosa was not smooth, and iodine in different areas was visible at 32–34 cm and 2–4 points from the incisor. The pathological report suggested adenocarcinoma. On October 10, 2016, the patient underwent cardiac cancer resection with colon interposition and regional lymph nodes cleaning *via* two thoracoabdominal incisions (upper abdominal median incision and right posterolateral thoracotomy). The tumor was located in the lesser curvature of the cardia, about 2 × 1 × 0.1 cm^3^ in size, invading the mucosa. The operation method for this case was the same as that of case 1. After the tumor was removed, the distribution of the colon blood vessels was observed. No variant arteriovenous was detected. The middle colon artery was ligated, the ascending colon, transverse colon, and part of the descending colon were freed, and the oral side of the transverse colon was disconnected. Subsequently, the ascending and descending colon was anastomosed end-to-side; the distal stomach and transverse colon were anastomosed end-to-side. Both of them used the Circular stapler (CDH25A, ETHINCON, USA). At 5-cm distal to the anastomotic opening of the stomach and transverse colon, the colon was ligated with two non-absorbable mousse suture and with staplers (TX60B, ETHICON, USA). And the mesocolon was preserved. Then, the patient was turned over, the right chest was opened to free and remove the thoracic esophagus, the colon was lifted to the chest, and end-to-side anastomosis was performed with the esophagus using the Circular stapler (CDH23A, ETHINCON, USA) Also, systematic lymph node dissection is necessary. The transplanted colon had an abundant blood supply. All anastomoses were end-to-side without tension, and the length of the transplanted colon was appropriate. The operation duration was 150 min.

The patient recovered well after the operation. Digestive tract angiography on October 15, 2016, showed that the esophageal mucosa was regular, the tube wall was soft, and the lumen was not narrowed or expanded (Figures 1D–F). Passage through the esophagus-colon anastomosis and colon-stomach anastomosis was smooth, with no abnormal channel. A close follow-up confirmed the absence of dysphagia, emptying disorders, malodor, or significant weight loss. His appetite was recovering, with no diarrhea or abdominal distension and rare acid reflux and heartburn occurrences. The last follow-up on December 21, 2021, showed no disease progression, slightly increased weight, and satisfactory quality of life.

## Discussion

This report described two cases of colon esophagoplasty that used the distal continual colon interposition method after some modifications. In both patients, digestion and absorption functions recovered well after the operation, and defecation was not affected. This method can be highly operable under total endoscopy, which is in line with the current development direction of minimally invasive treatment.

**Figure 2:**
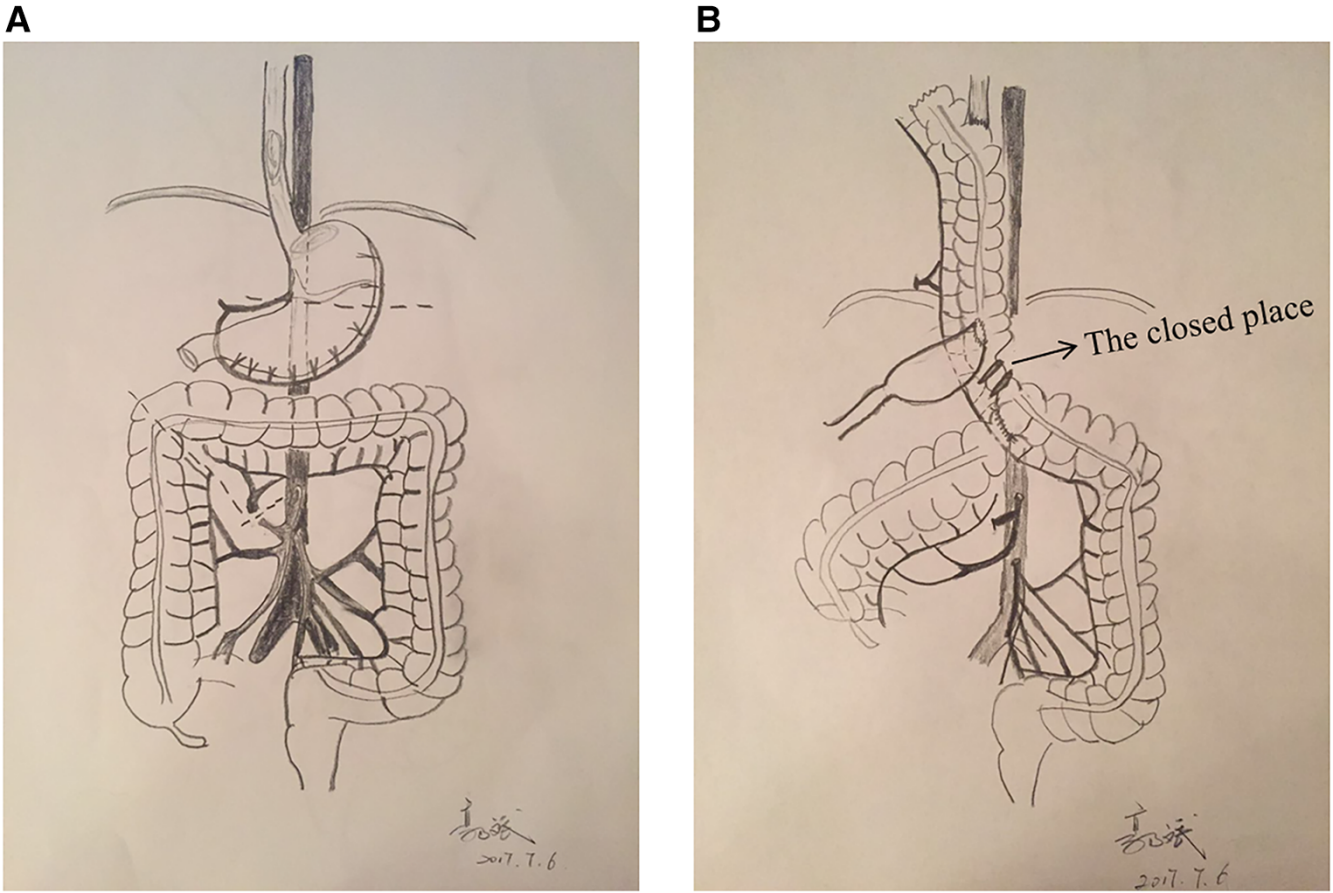
(**A**) Resection of the tumor with the proximal stomach, separation of the transverse colon and part of the descending colon, with a cut on the distal end of the transverse colon; (**B**) Location and method of each anastomosis. Arrow indicates the placement of the closure device. After resection of the thoracic esophagus, the transverse colon was lifted to the thoracic cavity for end-to-side anastomosis with the esophagus (pro-peristalsis). Then, the colon was successfully placed on the posterior mediastinal esophageal bed.

Esophageal replacement is a highly traumatic procedure affecting the neck, chest, abdomen, and upper and lower digestive tracts. Therefore, endoscopic resection of esophageal cancer has recently become the hotspot of research. Presently, the methods of esophageal reconstruction using various colon segments, such as ileum-transverse colon segment, ascending colon-transverse colon segment, and transverse colon-descending colon segment, have been reported (1, 10). Many operative methods of colon interposition proposed previously are based on the transplantation of the transverse colon, a part of the descending colon along the peristaltic direction with blood supply from the left colonic artery as the main stem of the left colonic artery has a constant origin, and the marginal blood vessels are complete (11). This report describes the interposition method without the complete detachment of the transplanted colon (Figure 2). After resection of the tumor, the stomach, transverse colon, and part of descending colon were separated, and a cut on the distal end of the transverse colon was made, retaining the left colonic artery and the integrity of the mesocolon (Figure 2A). End-to-side anastomosis of the ascending and descending colon and end-to-side anastomosis of the distal transverse gastric colon were performed. The transplanted colon was not disconnected completely but closed near the output end of the distal transverse gastrocolic anastomosis with a closure device [staplers add two non-absorbable mousse suture (TX60B, ETHICON, USA)] to block the passage of the food, while the blood supply of the intestinal canal and peripheral nerves was not disrupted (Figure 2B). As Wu Feng and his colleagues point that using more rows of staplers alone could not decrease the incidence of recanalization (12), so before the operation, the surgeon considered the possibility of colonic recanalization or transection (only ligating with silk suture), double non-absorbable mousse suture was used to ligate the intestinal canal and then close it with a stapler (TX60B, ETHICON, USA). However, this method still needs further validation. This proposed method was technically simpler than the traditional interposition and could prevent severe complications caused by mesangial vessel torsion. If the colon is cut off for reconstruction, the left mesocolic artery appears thin and easily tortuous when the transverse colon is lifted up through the right chest. And if the distal end of the transplanted colon is kept intact, the left colonic artery mesentery will be relatively fixed as the intestinal tube and difficult to twist. Also, these modifications decreased the operational difficulty and duration (to 140–150 min in the above cases). In conclusion, the procedure of colon interposition after this modification will be simpler and easier to control. It is convenient for minimally invasive surgery.

Compared to the stomach and jejunum, the colon has stable and uniform contraction, suitable for food delivery (8). As a graft, the colon could provide sufficient length (11). Therefore, using the colon as a graft instead of the esophagus leads to better food satisfaction and quality of life than other organs (9, 13). Moreover, pro-peristaltic anastomosis reduces the reflux *via* secondary peristalsis of the colon and prevents postoperative malodor (14). For patients with severe acid reflux symptoms before surgery, anastomosis can reduce the symptoms of acid reflux and heartburn after surgery (distal esophageal gastrostomy or jejunal interposition after subtotal gastrectomy) and can be further protected (15, 16). In addition, compared to the stomach, the structure and function of the colon are similar to that of the esophagus; colonic esophagus replacement has good acid resistance, and the incidence of reflux colitis is much lower than that of gastric esophagus replacement (16). In line with these advantages, both cases described above demonstrated satisfactory recovery, with no anastomotic stenosis or reflux colitis. During the follow-up period (>4 years for case 1 and >5 years for case 1), no heartburn, severe diarrhea, bloating, or malodor was reported, suggesting that the neurophysiological peristalsis function of the bowel transplant was preserved, and the possibility of mesocolon vessels torsion was lessened. However, patients with high surgical risk and low tumor prognosis should be cautiously examined before colon esophageal replacement surgery for malignant tumors.

The modified distal-continual colon interposition technique might have some advantages, such as a short operation duration and potential prevention of severe complications caused by mesocolon vessel torsion.

## Data Availability

The original contributions presented in the study are included in the article/Supplementary Material, further inquiries can be directed to the corresponding author/s.
